# Osteoporosis Therapy: Bone Modeling during Growth and Aging

**DOI:** 10.3389/fendo.2017.00046

**Published:** 2017-03-09

**Authors:** Toshihiro Sugiyama, Hiromi Oda

**Affiliations:** ^1^Department of Orthopaedic Surgery, Saitama Medical University, Saitama, Japan

**Keywords:** mechanical strain, bone modeling, osteoporosis, romosozumab, abaloparatide

## Mechanical Strain: The Primary Determinant of Bone Modeling

Remodeling-based bone resorption and formation are coupled on the same surface and contribute to calcium homeostasis, while modeling-based bone formation and resorption occur on different surfaces, such as during growth, to change skeletal shape; of importance, the aging skeleton can also include bone modeling (1, 2). The primary determinant of bone modeling is elastic deformation (strain) of the skeleton engendered by habitual physical activity, and accumulating evidence suggests that bones respond to mechanical loading to maintain their resultant strain (3–5). Not only osteocytes inside bone tissue but also bone-forming osteoblasts and bone-resorpting osteoclasts on the surface are responsive to mechanical signals (6, 7), and the modeling-based actions of osteoblasts and osteoclasts are not coupled.

Skeletal fragility depends on bone quality and quantity (8). The latter would be normally under the mechanical strain-related feedback control (3–5); Wolff’s law, established more than a century ago, confirms that mechanical environment plays a key role in controlling skeletal architecture (9), and the pattern of trabecular bone in the hip (Singh index) can be used for the evaluation of osteoporosis (10). In addition, this natural homeostatic system could work against mineral-related, but not collagen-related, impairment of bone material quality (11, 12).

Fragility fracture associated with osteoporosis is a common health problem during growth (13, 14) and aging (15, 16). Here, we provide a novel evidence-based insight into osteoporosis therapy from the viewpoint of bone modeling/remodeling, apart from approaches targeting bone formation/resorption and quality/quantity.

## Improving Skeletal Fragility by Bone Modeling

Normal bone growth is important not only for children but also for adults to prevent age-related fragility fracture (17, 18). Appropriate weight-bearing physical activities and calcium/vitamin D intakes support good bone acquisition during growth, but the treatment of severe pediatric osteoporosis has been highly limited. For instance, spontaneous fracture in children with physical disability, such as cerebral palsy, is a long-standing problem that affects their quality of life (19); the latest clinical practice guideline does not recommend regular use of bisphosphonates because their long-term effects on the growing skeleton are unclear (20). Although reduced mechanical loading is the major cause of skeletal fragility in individuals with physical disability, static weight-bearing physical activities as well as calcium/vitamin D supplementation are unlikely to prevent their fragility fractures (20). Fundamental rules of mechanical strain-related stimulus include strain rate as a key determinant of the stimulus and bones respond to dynamic, but not static, mechanical loading (21).

In the management of osteoporosis in children, it is important to note that their skeleton is different from the adult skeleton. Bone growth requires bone modeling that is predominantly influenced by mechanical environment and, therefore, cortical bone in lower limbs is very thin in children who cannot stand and walk. Consequently, an ideal method toward a cure for skeletal fragility during growth is pharmacologically stimulating mechanical strain-related bone modeling. Furthermore, bone modeling would also be useful for adults. One unmet need in elderly patients at high risk for fracture is to improve their skeletal fragility more rapidly; in this regard, bone modeling is superior to bone remodeling as with building reinforcement rather than rebuilding generally performed against earthquakes in Japan.

## Osteoporosis Agents and Bone Modeling

Most osteoporosis drugs are generally linked to bone remodeling rather than bone modeling, but it appears that some of them could be associated with bone modeling directly or indirectly (22). As discussed recently, daily or weekly treatment with teriparatide would stimulate modeling-based bone formation (11, 23). Bone modeling also seems to be stimulated by daily treatment with abaloparatide, an investigational agent (Table 1). Interestingly, daily subcutaneous injections of teriparatide (20 μg/day) and abaloparatide (80 μg/day) resulted in different effects on circulating markers of bone formation and resorption; teriparatide caused a rapid and sustained increase in bone formation followed by a delayed increase in bone resorption, while abaloparatide induced a relatively transient increase in bone formation with a less prominent increase in bone resorption (24). Of note, the effects of abaloparatide are partially similar to those of weekly subcutaneous injection of teriparatide (56.5 μg/week) used in Japan (11); the rapid but relatively transient increase in bone formation without the similar increase in bone resorption implies that daily treatment with abaloparatide stimulates more modeling-based bone formation compared to daily treatment with teriparatide. This is compatible with binding of abaloparatide to a G protein-dependent conformation of parathyroid hormone type 1 receptor with higher affinity but more transiently than teriparatide (25) and abaloparatide-induced increases in trabecular thickness and total area of cortical bone (26) and can reasonably explain greater increases in areal bone mineral density (BMD) at the femoral neck and total hip after daily treatment with abaloparatide versus teriparatide for 6 months (24). Accordingly, it is possible to speculate that, when teriparatide (20 μg/day) and abaloparatide (80 μg/day) are injected daily, the improvement of bone fragility could be faster by abaloparatide but better for longer duration by teriparatide (23), and abaloparatide rather than teriparatide might be more suitable for use in combination with denosumab (27). Nevertheless, both agents would not be used for children due to carcinogenicity in animals, though clinical experience with teriparatide has not presented such possibility in adults (28); to our knowledge, the use of teriparatide has been reported in a limited number of children with hypoparathyroidism (29), but not with osteoporosis.

**Table 1:** **Expected effects of anabolic osteoporosis agents on bone modeling**.

Anabolic osteoporosis agents	Bone modeling	Clinical status
**Parathyroid hormone**		
Teriparatide		
Daily (20 μg/day)^a^	+	Approval
Weekly (56.5 μg/week)^a^	+	Approval^b^
**Parathyroid hormone-related protein**		
Abaloparatide (daily, 80 μg/day)^a^	++	Phase 3
**Anti-sclerostin antibody**		
Romosozumab (monthly, 210 mg/month)^a^	+++	Phase 3

*^a^Subcutaneous injection*.

*^b^Japan and South Korea*.

In addition to teriparatide and abaloparatide, an increase in areal BMD over a prolonged period of time by treatment with osteoporosis drugs might partly result from bone modeling; examples could include denosumab (30, 31), strontium ranelate (32, 33), and investigational odanacatib (34–36). Although histomorphometric analysis of transiliac bone biopsies has not shown anabolic action of these agents, iliac bone is not a weight-bearing region as pointed out previously (33); their skeletal effects are site-specific and, therefore, iliac bone is unlikely to fully reflect each skeletal site (37).

## Stimulating Mechanical Strain-Related Bone Modeling

One promising therapeutic target for pharmacologically stimulating mechanical strain-related bone modeling is sclerostin (38–40), which is primarily secreted by osteocytes in the skeleton and inhibits the Wnt signaling pathway. On the basis of several lines of evidence, we have suggested that investigational anti-sclerostin antibodies, such as romosozumab (41, 42), possess the effect of mechanical strain-related stimulus that results in bone modeling (34, 43) (Table 1). This theory is supported by experimental findings that osteocyte sclerostin production is increased by skeletal disuse and decreased by skeletal loading (44, 45) and clinical data that circulating levels of sclerostin are higher after decreased physical activity (46, 47) and lower after increased physical activity (48, 49). High bone mass in patients with sclerostin deficiency is present throughout the skeleton, including non-weight-bearing regions, such as the face and skull (40), indicating that anti-sclerostin antibodies are not agents that decrease the mechanical strain threshold for bone modeling; treatment with an anti-sclerostin antibody is highly efficacious even under conditions with impaired physical activity (50–54), whereas enhancing skeletal response to physical activity cannot effectively improve bone fragility caused by reduced skeletal loading. Accordingly, treatment with an anti-sclerostin antibody is likely to strengthen the skeleton, without any specific direction in contrast to exercise, to prevent fall-related fractures such as in the hip (55).

Consequently, although most physically disabled individuals or elderly persons with severe osteoporosis would not perform dynamic exercise that results in bone modeling, anti-sclerostin antibodies are theoretically expected to contribute to a rapid improvement of their skeletal fragility by stimulating bone modeling. Sclerostin deficiency in humans leads to bone overgrowth progressively (40), and pharmacologic inhibition of sclerostin results in dose-related increases in areal BMD (41, 56) while monthly subcutaneous injection of romosozumab at a dose of 210 mg increased areal BMD at the lumbar spine and hip but not the radius in postmenopausal women (41), suggesting that the selected dose and interval are not enough for non-weight-bearing regions with higher levels of sclerostin expression and thus do not cause undesired bone overgrowth at the face or skull in adults. If the same treatment regimen is applied to different geographic regions, however, careful clinical practice might be needed in patients with very low body weight, such as in Asia. A higher dose and/or a shorter interval would be necessary for improving skeletal fragility in patients with physical disability, and clinical trials especially in children require careful investigation on their optimal doses and intervals; recent assessment did not find carcinogenicity risk of romosozumab (57).

## Conclusion

The therapeutic target of osteoporosis during childhood should be bone modeling, rather than bone remodeling, that is essential for skeletal growth. Bone modeling can also contribute to rapidly improving skeletal fragility in older adults toward goal-directed treatment for osteoporosis (58), though bone modeling in younger adults might have concerns from bone remodeling point of view. Anti-sclerostin antibodies, such as romosozumab, are promising drug candidates for stimulating mechanical strain-related bone modeling during growth and aging.

## Author Contributions

All authors listed have made substantial, direct, and intellectual contribution to the work and approved it for publication.

## Conflict of Interest Statement

The authors declare that the research was conducted in the absence of any commercial or financial relationships that could be construed as a potential conflict of interest.
